# The Effects of mHealth-Based Gamification Interventions on Participation in Physical Activity: Systematic Review

**DOI:** 10.2196/27794

**Published:** 2022-02-03

**Authors:** Linqi Xu, Hongyu Shi, Meidi Shen, Yuanyuan Ni, Xin Zhang, Yue Pang, Tianzhuo Yu, Xiaoqian Lian, Tianyue Yu, Xige Yang, Feng Li

**Affiliations:** 1 School of Nursing Jilin University Changchun China; 2 Faculty of Medicine and Life Sciences Hasselt University Hasselt Belgium; 3 School of Nursing Peking University Beijing China; 4 Department of Anaesthesia Bethune First Hospital of Jilin University Changchun China

**Keywords:** mobile health, gamification, physical activity, systematic review, mobile phone

## Abstract

**Background:**

It is well known that regular physical exercise has associated benefits; yet, participation remains suboptimal. Mobile health (mHealth) has become an indispensable medium to deliver behavior change interventions, and there is a growing interest in the gamification apps in mHealth to promote physical activity (PA) participation. Gamification could use game design elements (such as points, leaderboards, and progress bars), and it has the potential to increase motivation for PA and engagement. However, mHealth-based gamification interventions are still emerging, and little is known about the application status and efficacy of such interventions.

**Objective:**

This systematic review aims to investigate gamification apps in mHealth for improving PA levels and simultaneously summarize the impact of gamification interventions on PA participation.

**Methods:**

We searched PubMed, Scopus, Web of Science, Embase, CINAHL (EBSCO host), and IEEE Xplore from inception to December 20, 2020. Original empirical research exploring the effects of gamification interventions on PA participation was included. The papers described at least one outcome regarding exercise or PA participation, which could be subjective self-report or objective indicator measurement. Of note, we excluded studies about serious games or full-fledged games.

**Results:**

Of 2944 studies identified from the database search, 50 (1.69%) were included, and the information was synthesized. The review revealed that gamification of PA had been applied to various population groups and broadly distributed among young people but less distributed among older adults and patients with a disease. Most of the studies (30/50, 60%) combined gamification with wearable devices to improve PA behavior change, and 50% (25/50) of the studies used theories or principles for designing gamified PA interventions. The most frequently used game elements were goal-setting, followed by progress bars, rewards, points, and feedback. This review demonstrated that gamification interventions could increase PA participation; however, the results were mixed, and modest changes were attained, which could be attributed to the heterogeneity across studies.

**Conclusions:**

Overall, this study provides an overview of the existing empirical research in PA gamification interventions and provides evidence for the efficacy of gamification in enhancing PA participation. High-quality empirical studies are needed in the future to assess the efficacy of a combination of gamification and wearable activity devices to promote PA, and further exploration is needed to investigate the optimal implementation of these features of game elements and theories to enhance PA participation.

## Introduction

### Background

Regular physical activity (PA) correlates with varied physical and mental health benefits [1-4]. Guidelines reviewed by the Physical Activity Guidelines Advisory Committee recommended that even small increases in light-intensity PA participation can lead to health benefits [5-7]. However, despite proven benefits of PA participation, approximately one-third of the global adult population is insufficiently active and fails to fulfill the minimum PA guideline recommendations [8]. Moreover, an average adult spends approximately 8 hours of the day in sedentary mode [9], resulting in poor health outcomes, including an increased risk of cardiovascular disease and type 2 diabetes [10,11]. Therefore, innovative behavior change interventions are required to improve PA levels.

Mobile health (mHealth), as defined by the American Heart Association’s scientific statement, is “the use of mobile computing and communication technologies (eg, mobile phones, wearable devices) for health services and information” [12]. It has become an essential medium to bring about behavior change interventions and has demonstrated a promising role in improving PA levels [13]; for example, wearable activity trackers enable users to objectively monitor their PA levels when used in conjunction with a mobile app. The real-time feedback relating to daily steps from the app may provide ongoing support and motivation for maintaining healthy PA behavior [14].

Gamification is the use of game design elements (such as points, leaderboards, progress bars, and badges) in nongame contexts (such as management, education, marketing, and health care) to increase motivation and engagement [15]. There is a growing interest in the application of gamification in mHealth to promote healthy behavior change [16-19], especially in promoting PA levels [20]. For example, Patel et al [21] used gamification combined with social incentives to reward behaviors and finally increased PA among adults who were overweight and obese. As the concept of gamification is relatively new [15], empirical evidence is still emerging on the efficacy of gamification PA behavior change interventions.

To the best of our knowledge, no systematic review of quantitative studies has assessed the efficacy of gamification on PA behavior change. A systematic review in 2016 examined the amount and quality of empirical evidence for the efficacy of gamification on health and well-being [19]; however, the wide variability in gamification studies was limited in terms of the conclusions that could be drawn. Besides, the use of gamification in behavior change interventions is a young but rapidly growing research field; therefore, it would be timely to conduct a systematic review that combines all the empirical evidence related to the efficacy of gamification on PA participation.

### Aims

This systematic review aims to explore gamification apps in mHealth for improving PA levels and simultaneously summarize the effects of gamification interventions on PA participation. Specifically, this study aims to (1) determine the most commonly used type of mHealth (eg, wearable devices and mobile apps) to deliver PA gamification interventions, (2) describe the most commonly used game elements applied to mHealth for improving PA levels, (3) determine the behavior change theories used in PA gamification interventions, and (4) summarize the impact of gamification interventions on PA outcomes (including daily step counts and time spent in PA) and sedentary behavior.

## Methods

### Operationalizing Gamification

*Gamification* was defined and operationalized as the use of digital game elements in nongame contexts, which needs to be differentiated from creating immersive, full-fledged games as in serious games [15,22]. Serious games, sometimes referred to as *games with a purpose*, provide pure gaming experiences by creating a complete and immersive game (eg, augmented reality exergames such as Pokémon Go), whereas gamification attempts to affect users’ behavior and motivation through an experience reminiscent of games using game elements such as badges and points (eg, a wearable device combined with a mobile app used points and leaderboards to promote PA levels). However, the actual difference between the 2 concepts could be vague and highly subjective [22]. In cases where the concepts were indistinguishable, 3 investigators (LX, XY, and FL) discussed the issue and arrived at the final decision.

### Search Strategy

This systematic review was conducted according to the PRISMA (Preferred Reporting Items for Systematic Reviews and Meta-Analyses) guidelines and Cochrane guidelines for systematic reviews [23,24]. Candidate papers were searched in PubMed, Scopus, Web of Science, Embase, CINAHL (EBSCO host), and IEEE Xplore from inception to December 20, 2020. In addition, relevant papers from other systematic reviews were included. The search strategy used controlled vocabulary (Medical Subject Headings), natural language terms, and synonyms. The search keywords were *gamification*, *game element*, and *physical activity*. Multimedia Appendix 1 provides further details on the search strategy.

### Selection Criteria

The search results were imported into EndNote X9 (Clarivate) citation management software after removing the duplicates. All titles and abstracts of the candidate papers were screened by 2 investigators (LX and XZ). After the initial screening, 2 other investigators (MS and YP) independently reviewed the full text of the identified papers. Papers that fulfilled the following criteria were included in the systematic review:

1. Original empirical research, including qualitative and quantitative research (must be experimental research). Reviews (eg, systematic reviews, meta-analyses, narrative reviews, and scoping reviews), design documents, nonexperimental research, and protocols were excluded.
2. Peer-reviewed papers such as published papers, doctoral theses, and conference papers.
3. Full text is available in English.
4. Clearly specify gamification or the use of at least one game element. Research where gamification was only mentioned but not analyzed was excluded.
5. Gamification is delivered through digital devices (eg, PCs, tablets, smartphones, and wearable devices).
6. The purpose of gamification is to promote PA.
7. Serious games and full-fledged games (eg, video games as well as immersive virtual reality games and augmented reality exergames) were excluded.
8. The papers describe at least one outcome regarding exercise or PA participation, which could be subjective self-report or objective indicator measurement.
9. If there was a dispute over a reference, help from a third investigator was sought to resolve the issue and arrive at a final agreement.

### Study Quality

The quality of both the randomized controlled trials (RCTs) and quasi-experimental studies was evaluated by 2 authors (LX and MS). For all studies included in the systematic review, we performed a quality assessment using the Cochrane Effective Practice and Organization of Care Group controlled before-and-after studies risk-of-bias assessment recommendation [25]; this risk-of-bias assessment tool was equally applicable to the quality assessment of RCTs and quasi-experimental studies. A total of 9 risk-of-bias criteria, including selection, performance, and reporting, were used to assess the included studies for potential bias; besides, each criterion was rated as *low risk*, *high risk*, or *unclear risk.* We summarized the quality evaluation results using a diagram. Any disputes were resolved through discussion with a third investigator (Tianzhuo Y) to reach a final agreement.

### Data Extraction and Analysis

Working independently, 2 investigators (Tianyue Y and XL) extracted information from the selected studies into a prepared Microsoft Access form that was developed specifically for this systematic review. In cases of disagreement, the final decisions were taken after a discussion between the 2 investigators (Tianyue Y and XL). The recorded data in the systematic review included the name of the first author, publication year, country, study design, participant characteristics (population type, mean age, and percentage of the participants who were women), intervention characteristics (sample size, study setting, modality, and duration), gamification characteristics (game name, game elements, and theory used), and PA outcomes (PA measure, domains, and results). For the systematic review, the PA results comprised daily step counts, time spent in light PA (LPA), moderate PA (MPA), vigorous PA (VPA), moderate to vigorous PA (MVPA), percentage of goal reached, and PA motivation. Because of multiple definitions proposed for the term gamification, the subsequent classification methods of game elements were also divided. In this study, we used a combination of the taxonomy of game elements provided by Cugelman [26], Johnson et al [19], Lister et al [17], Sardi et al [16], and Vermeir et al [27]. The studies included in the systematic review had variations in study designs and insufficient data, which did not allow us to perform a meta-analysis. Therefore, we present the analysis of the PA outcomes and sedentary behavior in the form of a narrative review, with the results summarized in a table. Furthermore, we compared the inconsistencies of the intervention and gamification features between positive and negative studies to identify potential explanations.

## Results

### Search Results

**Figure 1:**
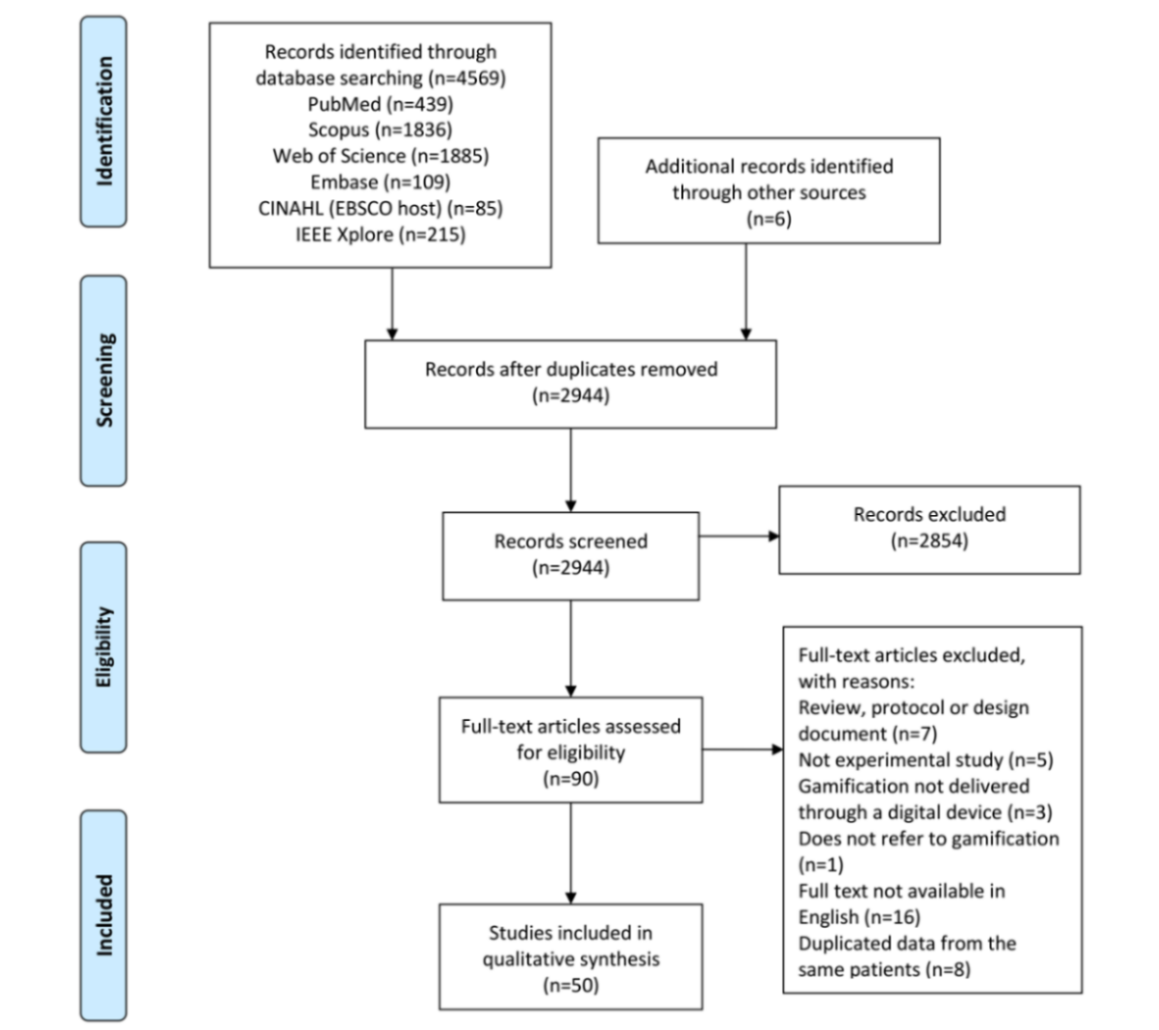
PRISMA (Preferred Reporting Items for Systematic Reviews and Meta-Analyses) flow diagram of search results.

A total of 4569 papers were identified through database searching, and an additional 6 papers were identified through other sources. Of these 4575 papers, after removal of duplicates, 2944 (64.35%) were screened by title or abstract. Of these 2944 papers, 2854 (96.94%) were excluded because they did not meet the inclusion and exclusion criteria, leaving 90 (3.06%) for full-text review. After careful evaluation, 44% (40/90) of the papers were excluded for the following reasons: 18% (7/40) were reviews, protocols, or design documents; 13% (5/40) were not experimental studies; 3% (1/40) did not refer to gamification; the gamification of 8% (3/40) was not delivered by means of a digital device; the full texts of 40% (16/40) were not available in English; and 20% (8/40) had duplicate data from the same patients. Finally, of the 90 studies, 50 (56%) were included and evaluated in our systematic review. Figure 1 shows the profile of the study selection.

### Study Characteristics

Multimedia Appendix 2 [21,28-76] presents the characteristics of all 50 papers included in our systematic review. The studies were published between 2013 and 2020, and 84% (42/50) were published after 2015, which indicated that research on using gamification to enhance PA was an emerging field and had a rapidly rising trend. The studies were distributed globally: 36% (18/50) in European countries, 24% (12/50) in the United States, 16% (8/50) in Asian countries, 10% (5/50) in Canada, 8% (4/50) in Australia, 4% (2/50) in Brazil, and 2% (1/50) in Singapore. The studies that were selected were primarily from two different types: RCTs (24/50, 48%) and quasi-experimental studies (26/50, 52%). Of the 26 quasi-experimental studies, 7 (27%) used a non-RCT design and 19 (73%) used a single-group pretest–posttest design. Both the RCTs and non-RCTs used a between-group design with 2, 3, 4, and 5 groups.

### Participant Characteristics

The systematic review included a total of 9977 participants, and evaluation was performed. Sample sizes varied from 7 to 3637 participants, with 84% (42/50) of the sample sizes consisting of <200 participants. When reported (45/50, 90%), participant types in 58% (26/45) of the studies were classified as low risk, including healthy adults (10/45, 22%), healthy adolescents (5/45, 11%), children (5/45, 11%), undergraduate students (3/45, 7%), and family (3/45, 7%), whereas participant types in 42% (19/45) of the studies were classified as high risk, including older adults (5/45, 11%); adults who were overweight or obese (4/45, 9%); insufficiently active people (3/45, 7%); and patients with rheumatoid arthritis (1/45, 2%), chronic obstructive pulmonary disease (1/45, 2%), childhood cancer (1/45, 2%), chronic back pain (1/45, 2%), coronary heart disease (1/45, 2%), ovarian cancer (1/45, 2%), and type 2 diabetes (1/45, 2%), indicating that the gamification of PA had been applied to a variety of population groups. The age of the participants ranged from 8 to 71 years, with the gamification interventions broadly distributed among young people but less distributed among older adults and patients with a disease. The proportion of women varied from 0% to 88%; of the 50 studies, 1 (2%) included only male participants and 7 (14%) did not report the gender ratio.

### Intervention Characteristics

Most of the study interventions (35/50, 70%) were conducted on the web, 12% (6/50) at homes, 8% (4/50) at schools, 4% (2/50) at workplaces, 4% (2/50) in communities, and 2% (1/50) in laboratories. The gamification of PA was delivered by means of several digital methods: mobile apps only (14/50, 28%), website only (6/50, 12%), activity monitors (eg, wristband and bracelet) only (7/50, 14%), website combined with activity monitors (9/50, 18%), and mobile apps combined with activity monitors (14/50, 28%), showing that most of the studies (30/50, 60%) combined gamification with wearable devices to improve PA behavior change. To be more specific, most of the wearable devices used in gamification were wrist worn (eg, Fitbit). The duration of the intervention ranged from 72 hours to 2 years; most (38/50, 76%) had no follow-up duration, indicating that further evaluations of PA gamified interventions are required to determine longer-term sustainability in the future.

### Gamification Characteristics

**Figure 2:**
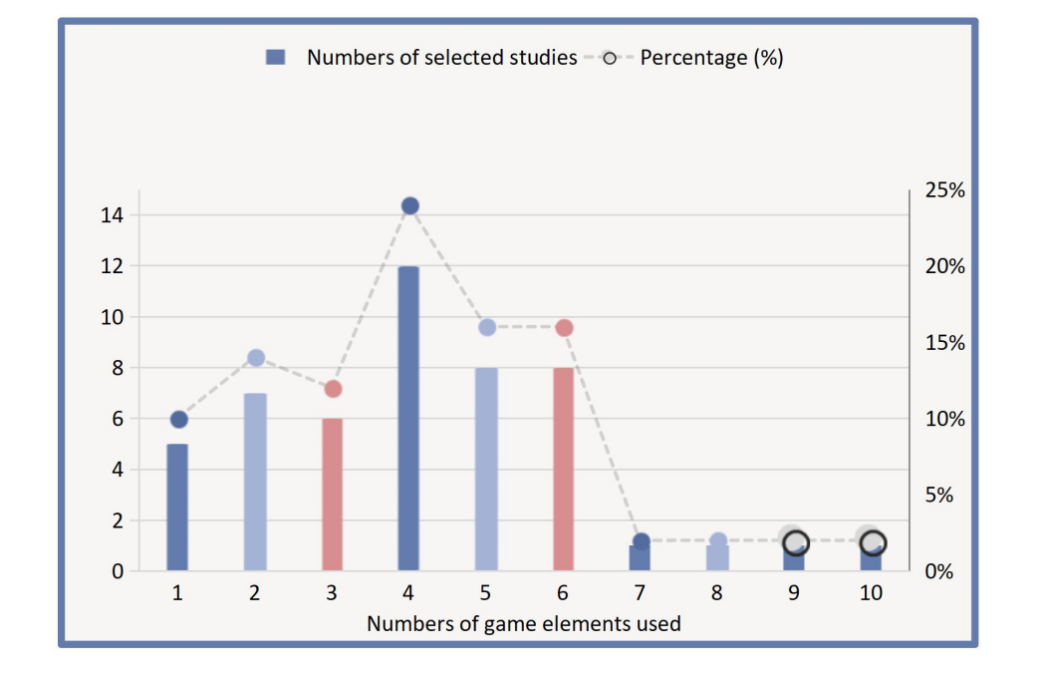
Number of game elements used in the selected studies.

Table 1 and Figure 2 show the gamification characteristics of the studies included in our systematic review. The number of game elements used in PA gamified interventions ranged from 1 to 10, with most including 5 game elements. The most frequently used game elements were goal-setting, followed by progress bars, rewards, points, and feedback.

Of the 50 studies, 25 (50%) used theories or principles for designing gamified PA interventions. As depicted in Table 2, self-determination theory (SDT) was used in 32% (8/25) of the studies, behavioral economics (BE) in 20% (5/25), social cognitive theory in 12% (3/25), theory of planned behavior in 12% (3/25), behavior change technology in 12% (3/25), the transtheoretical model in 12% (3/25), the Whole Person Wellness Model in 4% (1/25), theories of perceived value in 4% (1/25), fun theory in 4% (1/25), sociocognitive learning theory in 4% (1/25), and the health action process approach in 4% (1/25). Furthermore, most of the studies (22/25, 88%) used a single theory and 12% (3/25) used a combination of 2 theories.

**Table 1:** Type of game elements used in the selected studies (N=50).

Game elements		Values, n (%)
**Achievement and progression oriented**		
	Challenges	6 (12)
	Goal-setting	30 (60)
	Feedback	21 (42)
	Progress bars	26 (52)
	Points	22 (44)
	Levels	7 (14)
	Leaderboards	12 (24)
	Badges	6 (12)
	Rewards	25 (50)
**Social interaction oriented**		
	Competition	16 (32)
	Collaboration	16 (32)
	Social support	2 (4)
**Immersion oriented**		
	Story or theme	9 (18)
	Avatars	2 (4)

**Table 2:** Number of theories and principles used in the selected studies (N=25).

Theory	Values, n (%)
SDT^a^	8 (32)
BE^b^	5 (20)
SCT^c^	3 (12)
TPB^d^	3 (12)
BCT^e^	3 (12)
TTM^f^	3 (12)
WPWM^g^	1 (4)
Theories of perceived value	1 (4)
Fun theory	1 (4)
Sociocognitive learning theory	1 (4)
HAPA^h^	1 (4)

^a^SDT: self-determination theory.

^b^BE: behavioral economics.

^c^SCT: social cognitive theory.

^d^TPB: theory of planned behavior.

^e^BCT: behavior change technology.

^f^TTM: transtheoretical model.

^g^WPWM: Whole Person Wellness Model.

^h^HAPA: health action process approach.

### Assessment of Study Quality

**Figure 3:**
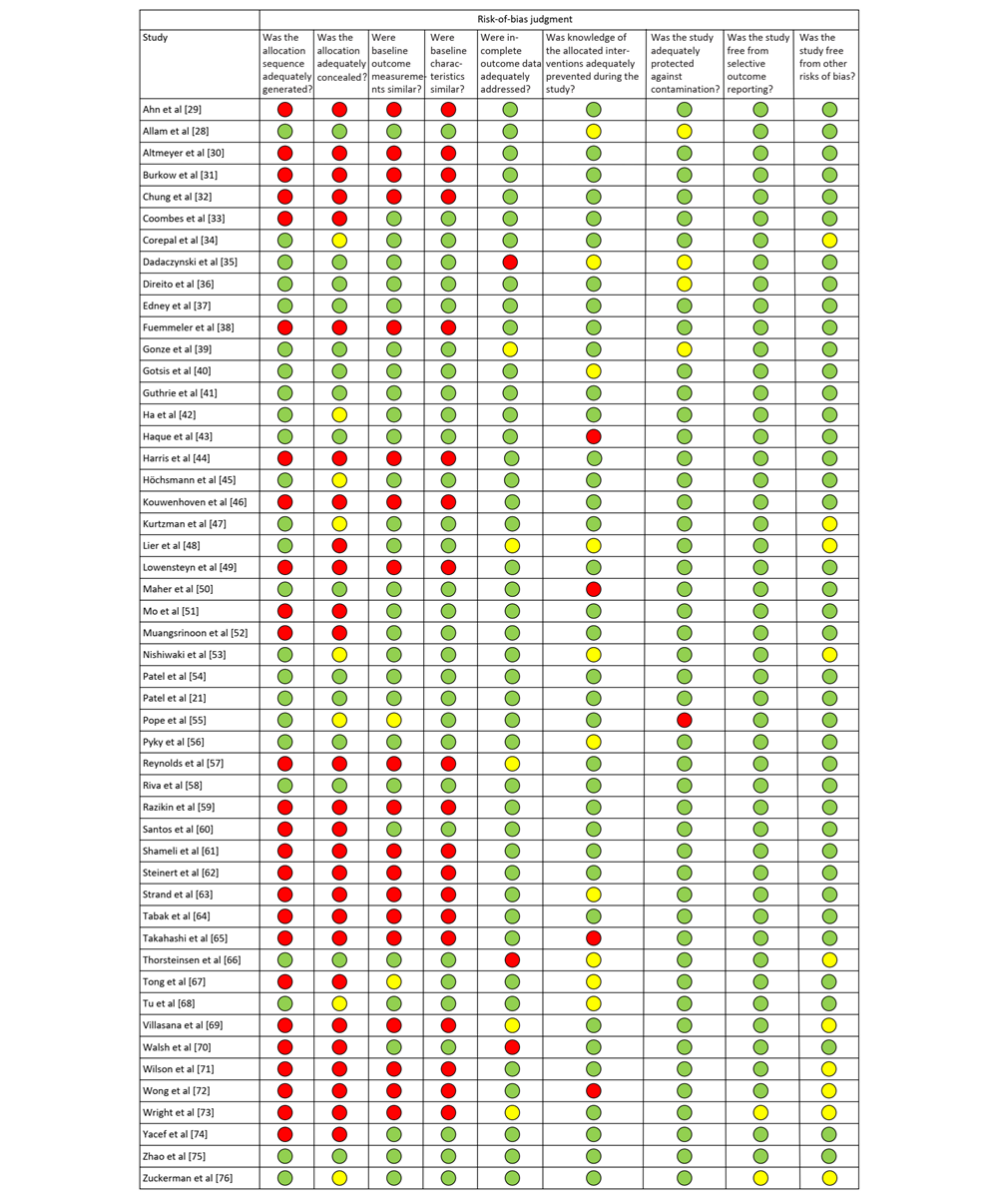
Risk-of-bias summary [21,28-76].

As mentioned in Figure 3, the quality of the 50 studies included in the systematic review was summarized using the Cochrane Effective Practice and Organization of Care Group risk-of-bias criteria. Generally, 58% (29/50) of the studies performed well, with at least 6 of the 9 evaluation criteria reported as low risk. As the RCTs and single-group pretest–posttest studies involved random sequence generation and allocation concealment, they were high risk. Furthermore, because 38% (19/50) of the studies had no control group, the applicable criteria relating to between-group comparisons were not fulfilled.

### Effects of Gamification on Outcome of PA

The PA behavior domains comprised daily step counts, time spent in LPA, MPA, VPA, and MVPA measured by objective activity monitors (34/50, 68%) or self-reported questionnaires (16/50, 32%). Multimedia Appendix 3 [21,28-76] provides a detailed summary of outcome measures, domains, and results for all included studies. Table 3 includes a summary of selected outcomes by study design. The controlled studies compared the differences between the intervention group and the control group, and the single-group studies simply compared the pre–post data in 1 group. Moreover, we compared the differences in intervention and gamification characteristics between positive and negative studies to identify potential reasons in Multimedia Appendix 4 [21,28-56,58-62, 64,66-72,74,76].

**Table 3:** Summary of selected outcomes by study design in the included studies (N=50).

Outcome and studies that assessed them (randomized controlled trials [study and effect])			Quasi-experimental studies (study and effect)		
			Nonrandomized controlled studies		Single-group (pre–post) studies
**Step counts (n=23)**					
	Corepal et al [34]^a^	Coombes et al [33]^a^		Ahn et al [29]^b^	
	Direito et al [36]^a^	Muangsrinoon et al [52]^c^		Altmeyer et al [30]^b^	
	Gonze et al [39]^a^	Santos et al [60]^c^		Chung et al [32]^d^	
	Höchsmann et al [45]^d^	Tong et al [67]^c^		Shameli et al [61]^b^	
	Kurtzman et al [47]^a^	Walsh et al [70]^a^		Tabak et al [64]^d^	
	Lier et al [48]^c^	—^e^		Takahashi et al [65]^d^	
	Nishiwaki et al [53]^c^	—		Wright et al [73]^d^	
	Patel et al [54]^c^	—		—	
	Patel et al [21]^c^	—		—	
	Pope et al [55]^a^	—		—	
	Tu et al [68]^c^	—		—	
**Time spent in overall PA^f^ (n=15)**					
	Allam et al [28]^b^	Mo et al [51]^c^		Altmeyer et al [30]^b^	
	Gotsis et al [40]^a^	—		Burkow et al [31]^d^	
	Haque et al [43]^c^	—		Harris [44]^b^	
	Maher et al [50]^a^	—		Lowensteyn et al [49]^b^	
	Nishiwaki et al [53]^c^	—		Razikin et al [59]^b^	
	Riva et al [58]^a^	—		Steinert et al [62]^b^	
	Thorsteinsen et al [66]^a^	—		Villasana et al [69]^a^	
	—	—		Wong et al [72]^b^	
**Time spent in LPA^g^ (n=7)**					
	Corepal et al [34]^a^	Mo et al [51]^c^		—	
	Dadaczynski et al [35]^c^	Yacef et al [74]^a^		—	
	Direito et al [36]^a^	—		—	
	Maher et al [50]^c^	—		—	
	Zuckerman et al [76]^c^	—		—	
**Time spent in MPA^h^ (n=6)**					
	Corepal et al [34]^a^	Mo et al [51]^c^		—	
	Dadaczynski et al [35]^a^	Yacef et al [74]^c^		—	
	Direito et al [36]^a^	—		—	
	Maher et al [50]^a^	—		—	
**Time spent in VPA^i^ (n=6)**					
	Corepal et al [34]^a^	Mo et al [51]^c^		—	
	Dadaczynski et al [35]^a^	Yacef et al [74]^c^		—	
	Direito et al [36]^a^	—		—	
	Maher et al [50]^a^	—		—	
**Time spent in** **MVPA^j^ (n=9)**					
	Corepal et al [34]^a^	Coombes et al [33]^c^		Fuemmeler et al [38]^b^	
	Direito et al [36]^a^	—		Kouwenhoven-Pasmooij et al [46]^b^	
	Edney et al [37]^a^	—		Wilson et al [71]^a^	
	Guthrie et al [41]^c^	—		—	
	Ha et al [42]^c^	—		—	
**Sedentary behavior (n=4)**					
	Direito et al [36]^c^	Yacef et al [74]^a^		Fuemmeler et al [38]^b^	
	Pyky et al [56]^a^	—		—	
**Percentage of goal reached (n=3)**					
	Patel et al [54]^c^	—		—	
	Patel et al [21]^c^	—		—	
	Zuckerman et al [76]^c^	—		—	
**PA** **motivation (n=3)**					
	Zhao et al [75]^c^	—		Reynolds et al [57]^d^	
	—	—		Strand et al [63]^b^	

^a^The between-group difference or the pre–post difference is not significant.

^b^The pre–post difference between groups is statistically significant.

^c^The difference between the intervention and control groups is statistically significant.

^d^There is a trend toward improvement, but the improvement is not significant.

^e^Not available.

^f^PA: physical activity.

^g^LPA: light physical activity.

^h^MPA: moderate physical activity.

^i^VPA: vigorous physical activity.

^j^MVPA: moderate to vigorous physical activity.

### Step Counts

Of the 50 included studies, 23 (46%) assessed the impact of PA gamification interventions on step counts. Of these 23 studies, 11 (48%) were RCTs, 5 (22%) were non-RCTs, and 7 (30%) were single-group studies. As depicted in Table 3, the results were quite consistent between the controlled studies and the single-group studies. The controlled studies (16/23, 65%) reported mixed results; 50% (8/16) [21,48,52-54,60,67,68] reported that the gamification interventions exerted a positive impact on step counts, 44% (7/16) [33,34,36,39,47,55,70] reported that no difference existed between the intervention and control groups for step counts, and 6% (1/16) [45] suggested a trend toward an increase in step counts after the gamification interventions, although the difference was not significant. The single-group studies (7/23, 30%) also reported mixed results; 43% (3/7) [29,30,61] reported that the pre–post difference within groups was statistically significant for step counts, whereas 57% (4/7) [32,64,65,73] reported that the pre–post difference was not significant.

### Time Spent in PA

#### Overview

Of the 50 included studies, 8 (16%) controlled studies and 8 (16%) single-group studies assessed the time spent in PA, as shown in Table 3, and the results were quite different between the controlled studies and the single-group studies. In the controlled studies, only 3 (3/8, 38%) [43,51,53] reported that the difference between the intervention and control groups was statistically significant. However, for the single-group studies, most of the studies (6/8, 75%) [30,44,49,59,62,72] demonstrated that the time spent in PA significantly increased after the gamification intervention. Only the study by Villasana et al [69] reported no trend toward improvement; the pre–post difference was not significant after the gamification intervention, and the study used just 1 game element (challenge) and did not use any theory (Multimedia Appendix 4).

We further examined the impact of gamification interventions on LPA, MPA, VPA, and MVPA.

#### Impact on LPA

Among the 50 included studies, time spent in LPA was assessed in 5 (10%) RCTs [34-36,50,76] and 2 (4%) non-RCTs [51,74] with mixed results. Of the 5 RCTs, 3 (60%) [35,50,76] showed that compared with the control groups, the intervention groups spent more time in LPA; however, the other 2 (40%) RCTs [34,36] reported that the differences between the intervention and control groups were not significant. In the non-RCTs, the study by Mo et al [51] reported that the gamification intervention exerted a positive impact on LPA, whereas the study by Yacef et al [74] reported no significant difference between the intervention and control groups. After comparing these 2 studies, we found that applying multiple and integrated gamification elements (>2 game elements) could be associated with positive effects on LPA.

#### Impact on MPA

Of the 50 included studies, 4 (8%) RCTs [34-36,50] and 2 (4%) non-RCTs [51,74] measured the time spent in MPA; the 4 (100%) RCTs [34-36,50] reported that the differences between the intervention and control groups were not significant, whereas the 2 (100%) non-RCTs [51,74] showed significant effects. The difference in the results between the RCTs and the non-RCTs could be attributed to the selection bias in the non-RCTs.

#### Impact on VPA

Among the 50 included studies, the outcomes of VPA were reported in 4 (8%) RCTs [34-36,50] and 2 (4%) non-RCTs [51,74]; of note, the results were different between these 2 types of studies. The RCTs [34-36,50] reported that no difference existed between the intervention and control groups for VPA; however, the non-RCTs [51,74] reported that the VPA was significantly increased in the intervention group compared with the control group.

#### Impact on MVPA

Of the 50 included studies, 9 (18%) studies reported the time spent in MVPA. Of these 9 studies, 6 (67%) were controlled studies [33,34,36,37,41,42] and 3 (33%) were single-group studies [38,46,71]; the results in both were mixed. In the 6 controlled studies, 3 (50%) [33,41,42] reported that the gamification intervention had positive effects on MVPA, whereas 3 (50%) [34,36,37] reported no significant difference between the intervention and control groups. In the 3 single-group studies, the pre–post difference between the groups for time spent in MVPA was significant in 2 (67%) studies [38,46] but not in the study by Wilson et al [71].

### Effects of Gamification on Sedentary Behavior

Sedentary behavior was reported as daily sitting time. Of the 50 included studies, 2 (4%) RCTs [36,56], 1 (2%) non-RCT [74], and 1 (2%) single-group study [38] reported this outcome; the results of the controlled studies were mixed, but the single-group study reported that the gamification intervention exerted a positive impact on sedentary behavior. In the 3 controlled studies, 1 (33%) RCT [36] reported that the intervention group spent less time in sitting compared with the control group, whereas the 2 (67%) other studies [56,74] reported no statistically significant differences between the intervention and control groups for daily sitting time. However, the single-group (pre–post) study [38] reported a significant decrease after the gamification intervention.

## Discussion

### Principal Findings

This study aims to offer a review of the gamification of PA. A total of 50 studies were included in the systematic review, suggesting that gamification in PA was still developing and lacked high-quality empirical research that could validate the efficacy of such interventions. The review revealed that gamification of PA had been applied to a variety of population groups and broadly distributed among young people but less distributed among older adults and patients with a disease. Most of the studies (30/50, 60%) combined gamification with wearable devices to improve PA behavior change. The most frequently used game elements were goal-setting, followed by progress bars, rewards, points, and feedback; besides, the most used theory in PA gamification was SDT. This systematic review revealed mixed findings for the efficacy of gamification interventions for improving PA participation and sedentary behavior. Both controlled studies and single-group studies reported mixed results on step counts, MVPA, and sedentary behavior. In addition, the controlled studies reported mixed results on time spent in LPA, MPA, and VPA. However, most of the single-group studies (6/8, 75%) revealed that gamified interventions might positively affect time spent in overall PA. Of note, these findings were limited because of the small number of studies.

### Gamification and mHealth

In the systematic review, the types of mHealth technologies used for delivering PA gamification interventions varied, with most of the studies using activity monitors (30/50, 60%), followed by mobile apps (28/50, 56%). To be more specific, most of the wearable devices used were wrist worn (eg, Fitbit). There is a growing interest in the use of wearable activity trackers to facilitate behavior management, when combined with the use of mobile apps; they might enhance users’ motivation for PA and help to better manage their health [77,78]. Wearable activity trackers could provide real-time feedback related to daily steps and energy expenditure by means of specifically designed algorithms or through health professionals [79,80], and when combined with gamification, they may markedly help in improving PA motivation and participation. However, there are few high-quality empirical studies. Thus, more empirical research is required in the future to explore the efficacy of a combination of gamification and wearable activity devices in promoting PA.

### Game Elements Used in PA Gamification

In the systematic review, the most frequently used game elements were achievement and progress oriented, such as goal-setting, progress bars, rewards, points, and feedback, which is consistent with previous reviews [26,27], suggesting that these were also the most frequently used elements in PA gamification interventions. Goal-setting (30/50, 60%) is a key technique for behavior change [26], and when it is combined with progress and feedback, it could markedly facilitate intrinsic motivation [81]. However, few scholars believe that rewards promote extrinsic motivation compared with intrinsic motivation; therefore, there may be a poor maintenance effect of the interventions [82].

The second most frequently used game elements in PA gamification interventions were social interaction oriented, such as competition and collaboration; these 2 elements increase users’ experience of fun and promote motivation for PA participation through social incentives. However, studies have demonstrated that different types and applications of social incentives might affect the efficacy of gamification interventions [21]. For example, gamification with collaboration among families led to significant increases in PA; however, the intervention was ineffective when conducted with participants who were previously unknown to each other [21,54]. Among such participants, competition became a more effective incentive method to promote PA. Therefore, future research needs to investigate the efficacy of gamification combined with different types of social incentives to promote PA participation.

### Gamification and Behavior Change Theories

In the systematic review, half of the studies used theories or principles for designing gamified PA interventions, and SDT (8/25, 32%) was the most commonly used theory, followed by BE (5/25, 20%). These findings were consistent with a previous systematic review [19]. SDT is a well-established motivation theory that has become a key framework for health behavior interventions because the motivation of individuals was recognized as the main factor driving behavior change [83]. However, intrinsic motivation or extrinsic motivation has different effects on behavior change, and existing research reveals that intrinsic motivation can promote not only behavior change in a more stable manner but also psychological and social well-being [19]. Hence, future research could consider applying gamification to promote intrinsic motivation to aid in improving PA participation.

The second most commonly used theory in PA gamification interventions was BE. In recent years, there has been a trend to use BE principles to guide interventions for improving PA [84]. From the perspective of BE principles, the decision to participate in PA is considered an investment in future health. An individual who is willing to *pay* the immediate costs of PA (eg, time and energy expenditure) to obtain health benefits in the future is regarded as having patient time preferences. We identified some predictable decision biases and chose interventions that persuade patients to choose a healthier decision (eg, participating in PA). Common BE principles embedded within PA gamification interventions included loss aversion, regret aversion, precommitment, and social norms [21,54].

### Effects of Gamification on PA and Sedentary Behavior

Overall, the evidence regarding the use of gamification to facilitate PA participation was inconclusive. Therefore, it is essential to consider potential explanations for the inconsistencies between the positive and negative studies. Regarding the time spent in overall PA, the positive impact of gamified interventions on PA was observed in 75% (6/8) of the single-group studies; these findings were consistent with a previous published systematic review [19], which reported that the positive impact of gamified interventions on PA was observed in 80% (8/10) of the studies. We further compared the differences in intervention and gamification characteristics between positive and negative studies. Of the 8 single-group studies, only 1 (13%) showed no trend toward improvement, and the pre–post difference was not significant in terms of the time spent in overall PA after the gamification intervention; the study used just 1 game element (challenge) and did not use any theory. These findings revealed that a combination of multiple game elements could be more effective for PA participation than a single game element, and gamification intervention using theory guidance could be more effective than a gamification intervention without any theory guidance. Furthermore, we tried to identify the appealing game features that could be associated with a positive effect; however, it is difficult to draw a definite conclusion because many studies have applied ≥2 gamification elements, and we cannot separate them to make a judgment. In addition, some of the studies [9,39] reported that participants liked the self-monitoring of progress and leader board aspects, which might be associated with the positive effect on PA outcomes. However, this should be interpreted with caution because of the heterogeneity of the selected studies.

Regarding the time spent in MPA and VPA, of the 50 included studies, 6 (12%) controlled studies measured the time spent in MPA and VPA and reported mixed results; the results differed between RCTs and non-RCTs. The bias in the non-RCTs could have potentially led to positive results. Our study reported mixed effects of gamification on daily sitting time. As far as we know, this is the first systematic review to report the impact of gamification on sedentary behavior; however, the results were limited because there were only a few high-quality empirical studies.

### Limitations

Our study includes several limitations. First, because of the variability and heterogeneity of the research interventions and results, the evidence might not be sufficiently strong to determine whether gamification effectively improves PA participation. Second, the studies included in the systematic review had variations in study design and insufficient data, which did not allow a meta-analysis. Third, although the population was diverse, the original articles had insufficient data, which prevented us from conducting a subgroup analysis based on the population. Fourth, related outcomes were measured immediately after the end of the intervention period, and the long-term effects of gamification in most studies were not observed; therefore, we did not summarize and synthesize the maintenance effect of the gamification interventions. Fifth, the differences in game elements, mHealth technology types, populations, and sample sizes among the included studies might be a major cause of the heterogeneity. Finally, most selected studies in our review were conducted using medical registry databases, which might suffer from an intrinsic risk of coding imprecision and incompleteness.

### Conclusions and Practical Implications

This study demonstrates that gamification interventions can increase PA participation; however, the results were mixed, and modest changes were obtained. This could be attributed to the heterogeneity across studies. Gamification combined with wearable activity trackers could help individuals to self-monitor progress and provide fun and motivation to promote health-related behavior change, especially in improving PA. Therefore, high-quality empirical studies are required in the future to examine the efficacy of a combination of gamification and wearable activity devices to promote PA. Gamification interventions generally have short-term effects, and ongoing contact by means of specifically designed algorithms and through health professionals could increase long-term adherence to PA participation. Hence, gamification combined with wearable activity devices has the potential to assist health professionals to provide ongoing support and motivation to patients who are physically inactive in terms of adherence to PA participation. Moreover, this study reveals that a combination of multiple game elements could be more effective for PA participation than a single game element, and a gamification intervention using theory guidance could be more effective than a gamification intervention without any theory guidance. The combination of different theories and different multiple game elements might produce different effects; hence, further exploration is required to explore the optimal implementation of these features of game elements and theories to improve PA participation. Furthermore, future empirical research on gamification should focus not only on the outcome of PA but also on the impact on sedentary behavior.

## Appendix

Multimedia Appendix 1 Full search strategy.

Multimedia Appendix 2 Summary descriptions of the studies included in the systematic review.

Multimedia Appendix 3 Summary of outcomes in the selected studies.

Multimedia Appendix 4 Comparing the differences of intervention and gamification characteristics between positive and negative studies.

## Abbreviations

BE: behavioral economics
LPA: light physical activity
mHealth: mobile health
MPA: moderate physical activity
MVPA: moderate to vigorous physical activity
PA: physical activity
PRISMA: Preferred Reporting Items for Systematic Reviews and Meta-Analyses
RCT: randomized controlled trial
SDT: self-determination theory
VPA: vigorous physical activity
