# Supplementary material for: The Effects of mHealth-Based Gamification Interventions on Participation in Physical Activity: Systematic Review
Source: JMIR Mhealth Uhealth. 2022 Feb 3;10(2):e27794. doi: 10.2196/27794 (PMC8855282; doi:10.2196/27794)
Supplement: Multimedia Appendix 1 [file mhealth_v10i2e27794_app1.docx]

**Multimedia Appendix 1: Literature Search Strategies**

We employed for each database full and truncated search terms in the following search string:

Gamification: (gamif* OR “game design element*” OR “game-design element*” OR “game element*” OR “game design interface pattern*” OR “game-design interface pattern*” OR“game interface element*” OR “game mechanic*” OR “game feature*” OR “game-like element*” OR “game-like feature*” OR “videogame element*”) AND

Physical activity: (physical activity* OR “exercises*” OR “physical exercise*” OR “acute exercise*” OR “isometric exercise*” OR “aerobic exercise*” OR “exercise training*” OR “physical activity behavior*” OR “physical activity lifestyle*”

Although the search strategy was the same for each database, suitable changes were made to accommodate for the different interfaces.

Example of a search strategy:

1. Database: Pubmed

Date searched: 20/11/2020

Hits: 439

(((((((((((((gasification) OR (game design elements)) OR (game design elements)) OR (game element)) OR (game design interface pattern)) OR (game-design interface pattern)) OR (game interface element)) OR (game mechanic)) OR (game feature)) OR (game like elements)) OR (game like features)) OR (videogame element))) AND ((((((((((physical activity) OR (exercises)) OR (Physical Exercise)) OR (Acute Exercise)) OR (Isometric Exercise)) OR (Aerobic Exercise)) OR (Exercise Training)) OR (physical activity behavior)) OR (physical activity lifestyle)))

2. Database: Scopus

Date searched: 20/11/2020

Hits: 1836

( ( TITLE-ABS-KEY ( gamification ) OR TITLE-ABS-KEY ( game AND design AND element ) OR TITLE-ABS-KEY ( game-design AND element ) OR TITLE-ABS-KEY ( game AND element ) OR TITLE-ABS-KEY ( game AND design AND interface AND pattern ) OR TITLE-ABS-KEY ( game-design AND interface AND pattern ) OR TITLE-ABS-KEY ( game AND interface AND element ) OR TITLE-ABS-KEY ( game AND mechanic ) OR TITLE-ABS-KEY ( game AND feature ) OR TITLE-ABS-KEY ( game-like AND element ) OR TITLE-ABS-KEY ( game-like AND feature ) OR TITLE-ABS-KEY ( videogame AND element ) ) ) AND ( ( TITLE-ABS-KEY ( physical AND activity ) OR TITLE-ABS-KEY ( exercises ) OR TITLE-ABS-KEY ( physical AND exercise ) OR TITLE-ABS-KEY ( acute AND exercise ) OR TITLE-ABS-KEY ( isometric AND exercise ) OR TITLE-ABS-KEY ( aerobic AND exercise ) OR TITLE-ABS-KEY ( exercise AND training ) OR TITLE-ABS-KEY ( physical AND activity AND behavior ) OR TITLE-ABS-KEY ( physical AND activity AND lifestyle ) ) )

3. Database: Web of Science

Date searched: 20/11/2020

Hits: 1885

主题: (gamification) OR 主题: (game design element) OR 主题: (game-design element) OR 主题: (game element) OR 主题: (game design interface pattern) OR 主题: (game-design interface pattern) OR 主题: (game interface element) OR 主题: (game mechanic) OR 主题: (game feature) OR 主题: (game-like element) OR 主题: (game-like feature) OR 主题: (videogame element) AND主题: (physical activity) OR 主题: (exercises) OR 主题: (physical exercise) OR 主题: (acute exercise) OR 主题: (isometric exercise) OR 主题: (aerobic exercise) OR 主题: (exercise training) OR 主题: (physical activity behavior) OR 主题: (physical activity lifestyle)

4. Database: Embase

Date searched: 20/11/2020

Hits: 109

gamification:ti,ab,kw OR 'game design element':ti,ab,kw OR 'game-design element':ti,ab,kw OR 'game element':ti,ab,kw OR 'game design interface pattern':ti,ab,kw OR 'game-design interface pattern':ti,ab,kw OR 'game interface element':ti,ab,kw OR 'game mechanic':ti,ab,kw OR 'game feature':ti,ab,kw OR 'game-like element':ti,ab,kw OR 'game-like feature':ti,ab,kw OR 'videogame element':ti,ab,kw AND 'physical activity':ti,ab,kw OR exercises:ti,ab,kw OR 'physical exercise':ti,ab,kw OR 'acute exercise':ti,ab,kw OR 'isometric exercise':ti,ab,kw OR 'aerobic exercise':ti,ab,kw OR 'exercise training':ti,ab,kw OR 'physical activity behavior':ti,ab,kw OR 'physical activity lifestyle':ti,ab,kw

5. Database: CINAHL (EBSCO host)

Host: EBSCOhost

Date searched: 20/11/2020

Hits: 85

Gamification OR game design element OR game-design element OR game element OR game design interface pattern OR game-design interface pattern OR game interface element OR game mechanic OR game feature OR game-like element OR game-like feature OR videogame element AND physical activity OR exercises OR physical exercise OR acute exercise OR isometric exercise OR aerobic exercise OR exercise training OR physical activity behavior OR physical activity lifestyle

6. Database: IEEE Xplore

Date searched: 20/11/2020

Hits: 215

(((((((((((((gasification) OR (game design elements)) OR (game design elements)) OR (game element)) OR (game design interface pattern)) OR (game-design interface pattern)) OR (game interface element)) OR (game mechanic)) OR (game feature)) OR (game like elements)) OR (game like features)) OR (videogame element))) AND ((((((((((physical activity) OR (exercises)) OR (Physical Exercise)) OR (Acute Exercise)) OR (Isometric Exercise)) OR (Aerobic Exercise)) OR (Exercise Training)) OR (physical activity behavior)) OR (physical activity lifestyle)))
