# Supplementary material for: The Effects of mHealth-Based Gamification Interventions on Participation in Physical Activity: Systematic Review
Source: JMIR Mhealth Uhealth. 2022 Feb 3;10(2):e27794. doi: 10.2196/27794 (PMC8855282; doi:10.2196/27794)
Supplement: Multimedia Appendix 2 [file mhealth_v10i2e27794_app2.docx]

**Multimedia Appendix 2. Summary descriptions of studies included in the systematic review.**

| Autho-r (year) | Count-ry | Study design | Participant Characteristics | | | | Intervention characteristics | | | Gamification characteristics | | |
| --- | --- | --- | --- | --- | --- | --- | --- | --- | --- | --- | --- | --- |
|  |  |  | Population  type | Mea-n age (SD) | %  Fema-le | Sam-ple size (N) | Study  Settin-g | Study  modality | Study duratio-n; follow-up | Game  name | Game  element | Theor-y  used |
| Allam et al. (2015) [28] | Switzerland | Between-subject (pre-post) design; 5 groups (Information sections only, social support sections, gaming sections, gaming plus social support, control) | Patients with RA | 57.95 | 46 | 155 | Home | Website | 2 months; 2 months | ONESELF | Points, leaderboards, badges, rewards | NS |
| Ahn et al. (2019) [29] | USA | Single group (pre-post) design | Children aged 9 to13 years | 11.24 | 61 | 67 | Laboratory | Activity monitor | 72 hours; none | Points-Based Reward Systems | Points, rewards, story/theme | SDT |
| Altmeyer et al. (2018) [30] | Germany | Single group (pre-post) design | Adults from fitness center and student council | 29.58 | 50 | 12 | Home | Mobile phone: app (Android), activity monitor | 269 days; none | Ggamified mobile app | Goal setting, progress, social interaction (social comparison) | SDT |
| Burkow et al. (2018) [31] | Norway | Single group (pre-post) design | Patients with COPD | 65.71 | 70 | 10 | Home | Tablet: app (Android), activity monitor: ProMove-3D activity sensor | 6 weeks; none | NS | Goal setting, feedback, rewards,social interaction (social support) | BCT |
| Chung et al. (2016) [32] | USA | Two group (pre-post) design; 2 groups(overweight/obese, healthy weight) | Overweight/obese adults, healthy weight adults | 19.76 | 67 | 12 | Home | Mobile phone: app (Twitter), activity monitor | 2 months; none | Tweeting to Health | Challenges, goal setting, feedback, rewards, social interaction | NS |
| Coombes et al. (2016) [33] | UK | Between-subject (pre-post) design; 2 groups(intervention, control) | Children aged 8 to 10 years | NS | 80 | NS | Travel to school | Activity monitor | 9 weeks; none | Beat the Street | Goal setting, feedback, progress, points, rewards, social interaction (social comparison) | Gamification theory |
| Corepal et al. (2019) [34] | Northern Ireland | Between-subject (pre-post) design; 2 groups(intervention, control) | Adolescents aged 12 to 14 years | NS | NS | 190 | School | Website, activity monitor | 22 weeks; 30 weeks | The StepSmart Challenge | Goal setting, feedback, leaderboards, rewards, social interaction (competition) | SDT |
| Dadaczynski et al.(2017) [35] | Germany | Between-subject (pre-post) design; 2 groups(intervention, control) | Healthy adults | 64.6 | 35 | 144 | Worksite setting | Website, activity monitor | 6 weeks; none | Healingo Fit | Chanllenges, goal setting, feedback, progress, points, levels, badges, leaderboards, social interaction (social comparison) | TPB, HAPA, socio-cognitive learning theory |
| Direito et al. (2015) [36] | New Zealand | Between-subject (pre-post) design; 3 groups(immersive app, nonimmersive app, control) | Insufficiently active healthy young people aged 14 to 17 years | 15.7 | 57 | 51 | Online | Mobile phone: app (Android and iOS) | 8 weeks; none | Apps for IMproving FITness (AIMFIT) | Progress, feedback, theme/story (immersive app-Zombies, Run);Progress, feedback(nonimmersive app-Get Running) | Self-regulatory behavior change techniques |
| Edney et al. (2020) [37] | Australia | Between-subject (pre-post) design; 3 groups(Active Team, self-monitoring app, waitlist control group) | Healthy adults | 41.3 | 74 | 444 | Online | Mobile phone: app, activity monitor | 100 days; 6 months | Active Team | Goal setting, social interaction, badges, progress, rewards | SCT |
| Fuemmeler et al. (2020) [38] | North Carolina | Single group (pre-post) design | Adolescent Survivors of Childhood Cancer | NS | NS | 15 | Online | Mobile phone: app, activity monitor | 8 weeks; none | Mila Blooms App | Points, badges, levels, story, goal setting, feedback, progress,rewards, challeges, social interaction | SCT, SDT |
| Gonze et al.(2020) [39] | Brazil | Between-subject (pre-post) design; 3 groups(smartphone app only, smartphone app + tailored messages, control) | Insufficiently active adults | 43 | 33 | 12 | Online | Mobile phone: app (Android and iOS) | 24 weeks,none | SMART | Challenges, goal setting, progress, badges, leaderboards, rewards, social interaction | NS |
| Gotsis et al. (2013) [40] | USA | Between-subject (crossover) design;2 groups(intervention, control) | Adults (egos) ages 44-88, and their family and friends (alters) ages 17-69 | 35.6 | 68 | 142 | Online | Website | 8 weeks; none | Wellness Partners | Points, rewards, story/theme, social interaction | NS |
| Guthrie et al. (2015) [41] | USA | Between-subject (pre-post) design;3 groups(intervention, active control, passive control) | Children aged 11to14 years | 12.7 | 88 | 182 | Online | Website, activity monitor | 6 weeks; none | Zamzee | Goal setting, feedback, rewards | NS |
| Ha et al.(2020) [42] | China | Between-subject (pre-post) design;2 groups(intervention, control) | Students | 14.4 | 69 | 667 | School | Activity monitor | 37 lessons;none | SELF-FIT | Feedback | SDT |
| Haque et al. (2020) [43] | Finland | Between-subject (pre-post) design;2 groups(intervention, control) | Office-based employees | 39 | 52 | 27 | Workplace | Mobile phone: app | 6 weeks; none | iGO mHealth app | Goal setting, progress, points, leaderboards, rewards, social interaction | SDT |
| Harris. (2020) [44] | UK | Single group (pre-post) design | People in community | NS | 60 | 722 | Community | Activity monitor | 6 weeks;1years, 2 years | Beat the Street | Goal setting, feedback, progress, points, rewards, social interaction (competition) | Gamification theory |
| Höchsmann et al. (2019) [45] | Switzerland | Between-subject (pre-post) design;2 groups(intervention, control) | Overweight type 2 diabetes patients | 57 | 47 | 36 | Online | Smartphone: app (iOS and Android) | 24 weeks,none | Mission: Schweinehund | Goal setting, feedback, rewards, story/theme | SDT |
| Kouwenhoven-Pasmooij et al. (2017) [46] | Netherlands | Single group (pre-post) design | Overweight and obese employees | 48.1 | 82 | 51 | Online | Website, activity monitor | 20 weeks,none | Movement Game | Goal setting, points, rewards, social interaction (competition) | NS |
| Kurtzman et al. (2018) [47] | USA | Between-subject (pre-post) design;2 groups (intervention, control) | Obese adults | 41.4 | 85.7 | 196 | Online | Website: Way to Health, smartphone(iOS) | 24 weeks;12weeks | LOSE IT | Goal setting, progress, points,levels,rewards, social interaction (collaboration), social incentives | BE |
| Lier et al. (2019) [48] | Germany | Between-subject (pre-post) design;2 groups(intervention, control) | Employees in worksite health promotion programs(WHPP) provider firm | 40.05 | 58 | 307 | Online | Mobilephone: app | 3 months;none | Provider H | Challenges, goal setting, social interaction (social comparison) | NS |
| Lowensteyn et al.(2019) [49] | Canada | Single group (pre-post) design | Permanent employees in a national company | NS | NS | 409 | Online | Website, activity monitor | 2 years; none | NS | Progress, social interaction (social comparison) | NS |
| Maher et al. (2015) [50] | Australia | Between-subject (pre-post) design;2 groups(intervention, control) | Insufficiently active adults | NS | 78 | 110 | Online | Mobile phone: app; activity monitor (pedometer) | 8 weeks, 12 weeks | Active Team | Goal setting, progress, feedback, awards, social interaction (collaboration) | TPB, fun theory |
| Mo et al. (2019) [51] | China | Between-subject (pre-post) design;2 groups(intervention, control) | Undergraduate Students | 20.74 | 52 | 52 | Online | Mobile phone: app(Wechat) | 7 weeks; none | NS | Points, leaderboards, rewards, social interaction (competetion),social incentives | TPB |
| Muangsrinoon et al.(2019) [52] | Thailand | Between-subject (post) design;2 groups(intervention, control) | Working Adults | NS | 62 | 60 | Online | Mobile phone: app, activity monitor: wristband | 8 weeks; none | NS | Goal setting, progress, feedback, points | NS |
| Nishiwaki et al. (2014) [53] | Japan | Between-subject (pre and post) design (crossover);2 groups(intervention, control) | Adults without chronic diseases | 31 | NS | 20 | Online | Mobile phone: app, activity monitor: Lifecorder EX | 6 weeks; 6 weeks | Yuuhokei | Goal setting, feedback, story/theme | NS |
| Patel et al.(2017) [54] | USA | Between-subject (post) design ;2 groups (intervention, control) | Family | 55.4 | 56 | 200 | Home | Website, activity monitor | 12 weeks; 12 weeks | BE FIT | Goal setting, progress, points,levels, rewards, social interaction (collaboration), social incentives | BE |
| Patel et al.(2019) [21] | USA | Between-subject (pre and post) design ;4 groups(support, collaboration,competition, control) | Overweight and Obese Adults | 39 | 29.1 | 602 | Online | Website, activity monitor | 24weeks; 12weeks | STEP UP | Goal setting, progress, points, levels, rewards, social interaction (support,collaboration,competition), social incentives | BE |
| Pope et al.(2018) [55] | Vermont | Between-subject (pre and post) design; 2 groups (intervention, control) | High school students | 17 | 71 | 75 | School | Website, activity monitor | 12 weeks; none | Camp Conquer | Avater | BE |
| Pyky et al. (2017) [56] | Finland | Between-subject (pre and post) design; 2 groups (intervention, control) | Young adolescent men | 17.8 | 0 | 496 | Online | Activity monitor (wrist-worn) | 6 months; none | MOPOrtal | Feedback, progress, rewards, social interaction(competition) | TTM |
| Reynolds et al. (2013) [57] | Canada | Single group (pre-post) design | NS | 36 | 53 | 15 | Home | Website | 1 month; none | Wii Fit | Feedback, points, progress, leaderboards, social interaction (competition), avater | NS |
| Riva et al. (2014) [58] | Switzerland | Between-subject (pre and post) design; 2 groups (intervention, control) | Chronic back pain (CBP) patients | NS | NS | 51 | Online | Website | 8 weeks, none | ONESELF | Feedback, points, leaderboard, rewards, social interaction (social comparison) | NS |
| Razikin et al.(2017) [59] | Singapore | Single group (pre-post) design | NS | 27.22 | 62.82 | 70 | Online | Mobile phone: app (Android) | 2 days, none | Zest! | Progress, points, leaderboards, social interaction | NS |
| Santos et al. (2019) [60] | Brazil | Between-subject (pre and post) design; 2 groups (intervention, control) | Older adults | 61.58 | 78 | 18 | Online | Mobile phone: app | 4 weeks; none | Trilhas | Social interaction (collaboration) | NS |
| Shameli et al. (2019) [61] | USA | Single group (pre-post) design | Users from app dataset | NS | NS | 3637 | Online | Smartphone app | 7 days; none | NS | Leaderboards, social interaction(competition) | NS |
| Steinert et al. (2018) [62] | Germany | Single group (pre-post) design | Older adults | 69 | 50 | 20 | Online | Mobile phone: app; activity monitor | 4 weeks; none | fMOOC | Goal setting, progress | NS |
| Strand et al. (2014) [63] | USA | Single group (pre-post) design | Older adults | NS | 87 | 46 | Community | Mobile phone: app | 8 weeks; 16 weeks | Wii Active | Goal setting, progress | WPWM, TTM |
| Tabak et al. (2020) [64] | Netherlands | Single group (pre-post) design | Older adults | 71 | 50 | 20 | Online | Mobile phone: app; activity monitor | 3 weeks; none | WordFit | Challenges, goal setting, progress, feedback, leaderboards, rewards, social interaction, story/theme | BCT |
| Takahashi et al. (2016) [65] | Japan | Single group (pre-post) design | Older adults | NS | 50 | 30 | Online | Mobile phone: app | 2 months; none | San-Poki | Social interaction | NS |
| Thorsteinsen et al.(2014) [66] | Norway | Between-subject (pre and post) design; 2 groups (intervention, control) | Healthy adults | 55.3 | NS | 21 | Online | Website | 12 week; none | NS | Goal setting, progress, feedback,badges, social interaction (competition) | NS |
| Tong et al. (2016) [67] | Canada | Between-subject (pre and post) design; 3 groups (FitPet, social, control) | NS | NS | 35 | 23 | Online | Mobile phone: app; Activity monitor: Fitbit | 6 weeks; none | FitPet | Goal setting, progress, social interaction, story/theme | TTM |
| Tu et al. (2018) [68] | China | Between-subject (pre and post) design; 2 groups (emotional value, social value) | Undergraduate students | 21.71 | 67 | 128 | Online | Mobile phone: app | 7 weeks; none | Walkup; WeChat Sports | Leaderboards, social interaction | theories of perceived value |
| Villasana et al. (2020) [69] | Portugal | Single group (pre-post) design | Teenagers | 15 | NS | 7 | Online | Mobile phone: app (Android) | 5 weeks; none | CoviHealth | Challenges | NS |
| Walsh et al. (2014) [70] | Canada | Between-subject (pre and post) design; 2 groups (intervention, control) | NS | 37.73 | 59 | 74 | Online | Website; activity monitoring (Fitbit Zip) | 30 days; none | StepCity | Leaderboards, social interaction | NS |
| Wilson et al. (2016) [71] | UK | Single group (pre-post) design | Adolescents with CHD | 15 | 56 | 27 | Online | Activity monitor (Fuelband) | 10 weeks; none | Fuelband | Goal setting, progress, points, rewards, social interaction | NS |
| Wong et al. (2020) [72] | China | Single group (pre-post) design | Parent-child pairs | NS | NS | 67 | Online | Mobile phone: app (iOS and Andriod) | 8 weeks; 5 months | Family Move app | Progress, points, levels, social interaction | SCT |
| Wright et al. (2020) [73] | USA | Single group (pre-post) design | Ovarian cancer survivors | 63 | NS | 24 | Online | Activity monitor | 12weeks; 12weeks | NS | Goal setting, points, levels, social interaction (collaboration) | BE |
| Yacef et al. (2018) [74] | Australia | Between-subject (pre and post) design; 2 groups (intervention, control) | Children aged 10–12 | NS | 45 | 59 | Online | Mobile phone: app; activity monitor (wrist-worn) | 1 month; none | iEngage | Goal setting, feedback | NS |
| Zhao et al. (2020) [75] | Canada | Between-subject (pre and post) design; 4 groups (gamified and personalized, gamified only, personalized only, control) | NS | 26.93 | 43 | 40 | Online | Mobile phone: app (iOS); activity monitor: smartwatches | 1 week; none | NS | Challenges, points, rewards, social interaction, story/theme | NS |
| Zuckerman et al. (2014) [76] | Israel | Between-subject (pre and post) design; 3 groups (quantified, points, leaderboard) | Undergraduate communications students | 23.39 | 75 | 59 | Online | Mobile phone: app (Android); activity monitor | 10 days; none | StepByStep | Goal setting, progress, feedback, points, rewards, social interaction | SDT |

Notes. NS= Not Specified; RA= Rheumatoid Arthritis; SDT=Self-Determination Theory; COPD= Chronic Obstructive Pulmonary Disease; BCT=Behavior Change Technologies; TPB= Theory of Planned Behavior; HAPA= the Health Action Process Approach; SCT=Social Cognitive Theory; BE= Behavioral Economics; TTM= the Transtheoretical Model; WPWM= the Whole Person Wellness Model; CHD, Coronary Heart Disease.
