# Supplementary material for: The Effects of mHealth-Based Gamification Interventions on Participation in Physical Activity: Systematic Review
Source: JMIR Mhealth Uhealth. 2022 Feb 3;10(2):e27794. doi: 10.2196/27794 (PMC8855282; doi:10.2196/27794)
Supplement: Multimedia Appendix 3 [file mhealth_v10i2e27794_app3.docx]

**Multimedia Appendix 3. Summary of outcomes in the selected studies.**

| Author (year) | Outcomes | | | |
| --- | --- | --- | --- | --- |
|  | Mearsure | Domain | Statistic | P |
| Allam et al. (2015)[28] | Questionnaire: Exercise Behaviors Scale | Time spent on PA in the last week (pre and post of social support sections plus gaming group) | [B]=3.39 | P^a^ =.02* |
| Ahn et al. (2019)[29] | Activity monitor: Fitbit Zip | Daily step counts (pre and post) | F = 5.32 | P^a^ =.0025* |
| Altmeyer et al. (2018) [30] | Activity monitor: Xiaomi Mi Band 1;  questionare: IPAQ-SF | Daily step counts (pre and post); MET-min/week (pre and post) | Z=-2.90; Z=-2.29 | P^a^=.004*; p^a^=.022* |
| Burkow et al. (2018)[31] | Self report: PA sessions | PA sessions in a week | NS | ↑ |
| Chung et al. (2016)[32] | Activity monitor: Fitbit Zip | Daily step counts(pre and post) | NS | ↑ |
| Coombes et al. (2016)[33] | Activity monitor: ActiGraph GT1M accelerometer | CPM (change between groups);  MVPA (min/day) (change between groups) | NS | P^a^=.823; P^a^=.020* |
| Corepal et al. (2019)[34] | Activity monitor: Fitbit Zip pedometers | Daily minutes of MVPA/LPA/MPA/VPA (min/day); Daily step counts (change between groups) | NS | - |
| Dadaczynski et al.(2017)[35] | Questionnaire: IPAQ-SF | Weekly minutes of LPA/MPA/VPA (min/week) (change between groups) | F=40.16; F=3.21; F=0.09 | P^a^<.001*; P^a^=.076; P^a^=.769 |
| Direito et al. (2015)[36] | Activity monitor: accelerometer (Actigraph GT1M);  Questionnaire: PAQ-A | Immersive:Daily step counts; Daily minutes of MVPA/LPA/MPA/VPA (min/day) (change between groups)  Nonimmersive: Daily step counts; Daily minutes of MVPA/LPA/MPA/VPA (min/day) (change between groups) | NS | P^a^=.98; P^a^=.91; P^a^=.98; P^a^=.90; P^a^=.99; P^a^=.99; P^a^=.96; P^a^=.99 |
| Edney et al. (2020)[37] | Activity monitor: wrist-worn pedometer (Zencro TW64S), Questionnaire: AAS | Objective daily minutes of MVPA(min/day); Self-reported weekly minutes of MVPA(min/day) (change between groups from baseline to 3 months) | F=0.32; F=1.04 | P ^b^ =.73;  P ^b^ =.36 |
| Fuemmeler et al. (2020)[38] | Activity monitor: pedometer | Daily minutes of MVPA(min/day); Percent of time in sedentary activity | NS | P ^a^ =.005 *;  P ^b^ =.021 * |
| Gonze et al.(2020)[39] | Activity monitor: triaxial accelerometer (ActiGraph GT3X+, MTI) | Daily step counts (post between groups) | NS | P ^a^ =.10 |
| Gotsis et al. (2013)[40] | Self report: one item(on average, how often do you exercise (minutes per day, days per week) | Weekly days of exercise (day/week) (post between groups) | F=2.56 | P ^a^ =.08 |
| Guthrie et al. (2015)[41] | Activity monitor: Zamzee activity meters | Daily minutes of MVPA(min/day) (change between groups)(passive/active) | 49%↑; 67%↑ | P ^a^< .001*;  P ^a^ < .001* |
| Ha et al.(2020)[42] | Activity monitor: ActiGraph GT3X+ and wGT3X-BT accelerometers worn at the hip | Percentage time spent in MVPA during PE (change between groups) | B = 4.00 | P ^b^ <.01 * |
| Haque et al. (2020)[43] | Self report: 7-point Likert scale | The score for increasing physical activity (post between groups) | NS | P ^a^ =.033* |
| Harris. (2020)[44] | Self report: single-item measure (In the past week, on how many days have you done a total of 30 min or more of physical activity?) | Weekly days of exercise (day/week) (pre and post in 1 year/2 years) | F= 35.049;  F = 14.916 | P ^a^ <.001*;  P ^a^ < .001* |
| Höchsmann et al. (2019)[45] | Activity monitor: Garmin Vivofit 2 activity wristband | Daily step counts | NS | ↑ |
| Kouwenhoven-Pasmooij et al. (2017)[46] | Activityn monitor: accelerometer (Activ8 system) | Daily minutes of MVPA(min/day) (pre and post) | B=1.03 | P ^a^ <.001* |
| Kurtzman et al. (2018)[47] | Activity monitor: smartphone recorded | Daily step counts (change between groups from basline to 24 weeks; from 24 weeks to 36 weeks) | NS | P ^a^ =.91;  P ^a^ =.62 |
| Lier et al. (2019)[48] | Activity monitor: smartphone recorded | Daily step counts (change between groups) | β = 1,139.36 | P ^a^⩽ .005* |
| Lowensteyn et al. (2019)[49] | Activity monitor: pedometer | METs (pre and post) | t=264 | P ^a^ <.05* |
| Maher et al. (2015)[50] | Questionare: AAS | Weekly minutes of LPA/MPA/VPA/overalll PA(min/week) (changes between groups from baseline to 8 weeks); Weekly minutes of LPA/MPA/VPA/overalll PA(min/week) (changes between groups from baseline to 20 weeks) | F=13.01; F=0.09; F=0.89; F=4.93; F=1.55; F=0.01; F=1.41; F=1.29 | P ^b^ <.001*;  P ^b^ =.77;  P ^b^ =.35;  P ^b^ =.03;  P ^b^ =.21;  P ^b^ =.94;  P ^b^ =.24;  P ^b^ =.26 |
| Mo et al. (2019)[51] | Quesqionare: IPAQ;  activity monitor: spreadsheet embedded in WeChat | Daily minutes of LPA/MPA/VPA (min/day); Weekly minutes of LPA/MPA/VPA (min/week);weekly sitting time(min/week); PA total score from IPAQ (changes between groups) | NS | P ^a^ =.735;  P ^a^ =.013*;  P ^a^ =.019*;  P ^a^ =.937;  P ^a^ <.001*;  P ^a^ <.001*;  P ^a^ =.005*;  P ^a^ <.001* |
| Muangsrinoon et al.(2019)[52] | Activity monitor: wristband (Xiaomi Band 2) | Daily step counts (post between groups) | t=5.68 | P ^a^ <.05* |
| Nishiwaki et al. (2014)[53] | Activity monitor: Lifecorder EX | Daily step counts (post between groups); Time > 3 METs (changes between groups) | NS | P ^a^ <.01*;  P ^a^ <.01* |
| Patel et al.(2017)[54] | Activity monitor: Fitbit | Daily step counts; proportion of participant-days achieving the step goal (post between groups from baseline to 12 weeks/post between groups from 12 weeks to 24 weeks) | NS | P ^a^ <.001*;  P ^a^ <.003;  P ^a^ <.001*;  P ^a^ <.001* |
| Patel et al.(2019)[21] | Activity monitor: Fitbit | Daily step counts (support/collaboration/competition) (changes between groups from baseline to 24 weeks); proportion of participant-days achieving the step goal (support/collaboration/competition) (changes between groups from baseline to 24 weeks) | NS | P ^a^ <.001*;  P ^a^ =.001;  P ^a^ <.001*;  P ^a^ <.001*;  P ^a^ <.001*;  P ^a^ <.001* |
| Pope et al.(2018)[55] | Activity monitor: Fitbit Flex | Daily step counts (post between groups) | NS | - |
| Pyky et al. (2017)[56] | Self-reported | Daily sitting time (hour/day) (change between groups) | NS | P ^a^ =.323 |
| Reynolds et al. (2013)[57] | Self-reported | NS | NS | ↑ |
| Riva et al. (2014)[58] | Questionnaire: Short Questionnaire to Assess Health-Enhancing Physical Activity | Weekly time spent on physical exercise (hour/week) | d=0.36 | P ^a^ >.05 |
| Razikin et al.(2017)[59] | Questionnaire | Questionnaire scores (pre and post) | F=13.79 | P ^a^ <.001* |
| Santos et al. (2019)[60] | Activity monitor: mobile phone recorded | Weekly step counts (change between groups) | η2=.19 | P ^a^ =.01* |
| Shameli et al. (2019)[61] | Activity monitor: smartphone accelerometers | Daily step counts | D=1400 | P ^a^ <.05* |
| Steinert et al. (2018)[62] | Activity monitor: Garmin vívofit | Daily physical activity time (min/day)  (pre and post) | T = − 2.274 | P ^a^ <.05* |
| Strand et al. (2014)[63] | Self-reported physical activity participation (Do you currently engage in regular physical activity?) | Propotion of participants from inactive to active(1-25weeks; 8-25 weeks) | NS | P ^a^ =.001*;  P ^a^ =.014* |
| Tabak et al. (2020)[64] | Activity monitor: FitBit | Daily step counts (pre and post) | NS | ↑ |
| Takahashi et al. (2016)[65] | Self report | Walking distance, steps increase | NS | ↑ |
| Thorsteinsen et al.(2014)[66] | Daily self-report | Weekly minutes of PA | F=0.264 | P ^a^ >.05 |
| Tong et al. (2016)[67] | Activity monitor: Fitbit | Daily step counts (change between groups) (social and controll) | NS | P^b^ =.03* |
| Tu et al. (2018)[68] | Activity monitor: app recorded | Daily step counts (post between groups) | t=5.737 | P ^a^ <.001* |
| Villasana et al. (2020)[69] | Activity monitor: app recorded | Frequency of physical exercise per week (pre and post) | NS | P ^a^ =.486 |
| Walsh et al. (2014)[70] | Activity monitor: Fitbit Zip | Total step counts (post between groups) | t = 1.3386 | P ^a^ =.09 |
| Wilson et al. (2016)[71] | Activity monitor: Fuelband | Daily minutes of MVPA(min/week) (pre and post) | NS | P=.335 |
| Wong et al. (2020)[72] | Questionnaire: IPAQ-SF | Weekly PA level expressed in MET-minutes/week (pre and post at 1 month/6 months) | d=0.31; d=0.30 | P ^a^ = .030*;  P ^a^ = .042* |
| Wright et al. (2020)[73] | Activity monitor | Daily step counts | NS | ↑ |
| Yacef et al. (2018)[74] | Activity monitor: research-grade accelerometer (Geneactiv) | Percentages of time spent in LPA/MPA/VPA and sedentary (pre and post in the intervention group) | NS | P ^a^ =.1295;  P ^a^ =.0035*;  P ^a^ =.0175*;  P ^a^ =.7829 |
| Zhao et al. (2020)[75] | Activity monitor: Android ActivityRecognition and Google Fit API; Questionare | Overall motivation | F=22.49 | P ^a^ <.001* |
| Zuckerman et al. (2014)[76] | Activity monitor: custom algorithm | Daily walking time (min); Daily percent of goal reached ( post between groups) | NS | P ^a^ <.05* |

Notes. NS= Not Specified; PA= Physical Activity; IPAQ-SF= International Physical Activity Questionnaire-short form; IPAQ=International Physical Activity Questionnaire; METs=Metabolic Equivalent Times; CPM=Average counts per minute; MVPA=Moderate-to-Vigorous Physical Activity; LPA=Light physical activity; MPA=Moderate Physical Activity; VPA=Vigorous Physical Activity; PAQ-A= Physical Activity Questionnaire for Adolescents; PE= Physical Education; AAS=Active Australia Survey; IPAQ-SF= The International Physical Activity Questionnaire-Short Form-Chinese version; API=Application Programming Interface.

^a^ P-values for the main effect of time;

^b^ P-values for the interaction of group and time;

*Significantly statistics.
