# Supplementary material for: The Effects of mHealth-Based Gamification Interventions on Participation in Physical Activity: Systematic Review
Source: JMIR Mhealth Uhealth. 2022 Feb 3;10(2):e27794. doi: 10.2196/27794 (PMC8855282; doi:10.2196/27794)
Supplement: Multimedia Appendix 4 [file mhealth_v10i2e27794_app4.docx]

**Multimedia Appendix 4 Comparing the differences of intervention and gamification characteristics between positive and negative studies**

**Section 1. The effects on step counts**

| **Randomized controlled trials for step counts** | | | | |
| --- | --- | --- | --- | --- |
| Study | Effect | Study modality | Game element | Theory  used |
| Lier et al.[48] | ** | Mobilephone: app | Challenges, goal setting, social interaction (social comparison) | NS |
| Nishiwaki et al.[53] | ** | Mobile phone: app, activity monitor: Lifecorder EX | Goal setting, feedback, story/theme | NS |
| Patel et al.(2017)[54] | ** | Website, activity monitor | Goal setting, progress, points,levels, rewards, social interaction (collaboration), social incentives | BE |
| Patel et al.(2019) [21] | ** | Website, activity monitor | Goal setting, progress, points, levels, rewards, social interaction (support,collaboration,competition), social incentives | BE |
| Tu et al.[68] | ** | Mobile phone: app | Leaderboards, social interaction | theories of perceived value |
| Corepal et al.[34] | ^ | Website, activity monitor | Goal setting, feedback, leaderboards, rewards, social interaction (competition) | SDT |
| Direito et al.[36] | ^ | Mobile phone: app (Android and iOS) | Chanllenges, goal setting, feedback, progress, points, levels, badges, leaderboards, social interaction (social comparison) | TPB, HAPA, socio-cognitive learning theory |
| Gonze et al.[39] | ^ | Mobile phone: app (Android and iOS) | Challenges, goal setting, progress, badges, leaderboards, rewards, social interaction | NS |
| Höchsmann et al.[45] | ↑ | Smartphone: app (iOS and Android) | Goal setting, feedback, rewards, story/theme | SDT |
| Kurtzman et al.[47] | ^ | Website: Way to Health, smartphone(iOS) | Goal setting, progress, points, levels, rewards, social interaction (collaboration), social incentives | BE |
| Pope et al.[55] | ^ | Website, activity monitor | Avater | BE |
| **Non-randomized controlled studies for step counts** | | | | |
| Muangsrinoon et al.[52] | ** | Mobile phone: app, activity monitor: wristband | Goal setting, progress, feedback, points | NS |
| Santos et al.[60] | ** | Mobile phone: app | Social interaction (collaboration) | NS |
| Tong et al.[67] | ** | Mobile phone: app; Activity monitor: Fitbit | Goal setting, progress, social interaction, story/theme | TTM |
| Coombes et al.[33] | ^ | Activity monitor | Goal setting, feedback, progress, points, rewards, social interaction (social comparison) | Gamification theory |
| Walsh et al.[70] | ^ | Website; activity monitoring (Fitbit Zip) | Leaderboards, social interaction | NS |
| **Single group (pre-post) studies for step counts** | | | | |
| Ahn et al.[29] | * | Activity monitor | Points, rewards, story/theme | SDT |
| Altmeyer et al.[30] | * | Mobile phone: app (Android), activity monitor | Goal setting, progress, social interaction (social comparison) | SDT |
| Shameli et al.[61] | * | Smartphone app | Leaderboards, social interaction(competition) | NS |
| Chung et al.[32] | ↑ | Mobile phone: app (Twitter), activity monitor | Challenges, goal setting, feedback, rewards, social interaction | NS |
| Tabak et al.[64] | ↑ | Mobile phone: app; activity monitor | Challenges, goal setting, progress, feedback, leaderboards, rewards, social interaction, story/theme | BCT |

**Section 2 The effects on time spent in physical activity**

**Time spent in overall PA**

| **Randomized controlled trials for time spent in overall PA** | | | | |
| --- | --- | --- | --- | --- |
| Study | Effect | Study modality | Game element | Theory  used |
| Haque et al.[43] | ** | Mobile phone: app | Goal setting, progress, points, leaderboards, rewards, social interaction | SDT |
| Nishiwaki et al.[53] | ** | Mobile phone: app, activity monitor: Lifecorder EX | Goal setting, feedback, story/theme | NS |
| Allam et al.[28] | * | Website | Points, leaderboards, badges, rewards | NS |
| Gotsis et al.[40] | ^ | Website | Points, rewards, story/theme, social interaction | NS |
| Maher et al.[50] | ^ | Mobile phone: app; activity monitor (pedometer) | Goal setting, progress, feedback, awards, social interaction (collaboration) | TPB, fun theory |
| Riva et al.[58] | ^ | Website | Feedback, points, leaderboard, rewards, social interaction (social comparison) | NS |
| Thorsteinsen et al.[66] | ^ | Website | Goal setting, progress, feedback,badges, social interaction (competition) | NS |
| **Non-randomized controlled studies for time spent in overall PA** | | | | |
| Mo et al. [51] | ** | Mobile phone: app(Wechat) | Points, leaderboards, rewards, social interaction (competetion),social incentives | TPB |
| **Single group (pre-post) studies for time spent in overall PA** | | | | |
| Altmeyer et al.[30] | * | Mobile phone: app (Android), activity monitor | Goal setting, progress, social interaction (social comparison) | SDT |
| Harris.[44] | * | Activity monitor | Goal setting, feedback, progress, points, rewards, social interaction (competition) | Gamification theory |
| Lowensteyn et al.[49] | * | Website, activity monitor | Progress, social interaction (social comparison) | NS |
| Razikin et al.[59] | * | Mobile phone: app (Android) | Progress, points, leaderboards, social interaction | NS |
| Steinert et al.[62] | * | Mobile phone: app; activity monitor | Goal setting, progress | NS |
| Wong et al.[72] | * | Mobile phone: app (iOS and Andriod) | Progress, points, levels, social interaction | SCT |
| Burkow et al.[31] | ↑ | Tablet: app (Android), activity monitor: ProMove-3D activity sensor | Goal setting, feedback, rewards,social interaction (social support) | BCT |
| Villasana et al.[69] | **^** | Mobile phone: app (Android) | **Challenges** | **NS** |

**Time spent in LPA**

| **Randomized controlled trials for time spent in LPA** | | | | |
| --- | --- | --- | --- | --- |
| Study | Effect | Study modality | Game element | Theory  used |
| Dadaczynski et al.[35] | ** | Website, activity monitor | Chanllenges, goal setting, feedback, progress, points, levels, badges, leaderboards, social interaction (social comparison) | TPB, HAPA, socio-cognitive learning theory |
| Maher et al.[50] | ** | Mobile phone: app; activity monitor (pedometer) | Goal setting, progress, feedback, awards, social interaction (collaboration) | TPB, fun theory |
| Zuckerman et al.[76] | ** | Mobile phone: app (Android); activity monitor | Goal setting, progress, feedback, points, rewards, social interaction | SDT |
| Corepal et al.[34] | ^ | Website, activity monitor | Goal setting, feedback, leaderboards, rewards, social interaction (competition) | SDT |
| Direito et al.[36] | ^ | Mobile phone: app (Android and iOS) | Progress, feedback, theme/story (immersive app-Zombies, Run);Progress, feedback (nonimmersive app-Get Running) | Self-regulatory behavior change techniques |
| **Non-randomized controlled studies for time spent in LPA** | | | | |
| Mo et al.[51] | ** | Mobile phone: app (Wechat) | Points, leaderboards, rewards, social interaction (competetion),social incentives | TPB |
| Yacef et al.[74] | ^ | Mobile phone: app; activity monitor (wrist-worn) | Goal setting, feedback | NS |

**Time spent in MPA**

| **Randomized controlled trials for time spent in MPA** | | | | |
| --- | --- | --- | --- | --- |
| Study | Effect | Study modality | Game element | Theory  used |
| Corepal et al.[34] | ^ | Website, activity monitor | Goal setting, feedback, leaderboards, rewards, social interaction (competition) | SDT |
| Dadaczynski et al.[35] | ^ | Website, activity monitor | Chanllenges, goal setting, feedback, progress, points, levels, badges, leaderboards, social interaction (social comparison) | TPB, HAPA, socio-cognitive learning theory |
| Direito et al.[36] | ^ | Mobile phone: app (Android and iOS) | Progress, feedback, theme/story (immersive app-Zombies, Run);Progress, feedback(nonimmersive app-Get Running) | Self-regulatory behavior change techniques |
| Maher et al.[50] | ^ | Mobile phone: app; activity monitor (pedometer) | Goal setting, progress, feedback, awards, social interaction (collaboration) | TPB, fun theory |
| **Non-randomized controlled studies for time spent in MPA** | | | | |
| Mo et al.[51] | ** | Mobile phone: app(Wechat) | Points, leaderboards, rewards, social interaction (competetion),social incentives | TPB |
| Yacef et al.[74] | ** | Mobile phone: app; activity monitor (wrist-worn) | Goal setting, feedback | NS |

**Time spent in VPA**

| **Randomized controlled trialss for time spent in VPA** | | | | |
| --- | --- | --- | --- | --- |
| Study | Effect | Study modality | Game element | Theory  used |
| Corepal et al.[34] | ^ | Website, activity monitor | Goal setting, feedback, leaderboards, rewards, social interaction (competition) | SDT |
| Dadaczynski et al.[35] | ^ | Website, activity monitor | Chanllenges, goal setting, feedback, progress, points, levels, badges, leaderboards, social interaction (social comparison) | TPB, HAPA, socio-cognitive learning theory |
| Direito et al.[36] | ^ | Mobile phone: app (Android and iOS) | Progress, feedback, theme/story (immersive app-Zombies, Run);Progress, feedback(nonimmersive app-Get Running) | Self-regulatory behavior change techniques |
| Maher et al.[50] | ^ | Mobile phone: app; activity monitor (pedometer) | Goal setting, progress, feedback, awards, social interaction (collaboration) | TPB, fun theory |
| **Non-randomized controlled studies for time spent in VPA** | | | | |
| Mo et al.[51] | ** | Mobile phone: app(Wechat) | Points, leaderboards, rewards, social interaction (competetion),social incentives | TPB |
| Yacef et al.[74] | ** | Mobile phone: app; activity monitor (wrist-worn) | Goal setting, feedback | NS |

**Time spent in MVPA**

| **Randomized controlled trialss for time spent in MVPA** | | | | |
| --- | --- | --- | --- | --- |
| Study | Effect | Study modality | Game element | Theory  used |
| Guthrie et al.[41] | ** | Website, activity monitor | Goal setting, feedback, rewards | NS |
| Ha et al.[42] | ** | Activity monitor | Feedback | SDT |
| Corepal et al. [34] | ^ | Website, activity monitor | Goal setting, feedback, leaderboards, rewards, social interaction (competition) | SDT |
| Direito et al.[36] | ^ | Mobile phone: app (Android and iOS) | Progress, feedback, theme/story (immersive app-Zombies, Run);Progress, feedback(nonimmersive app-Get Running) | Self-regulatory behavior change techniques |
| Edney et al. [37] | ^ | Mobile phone: app, activity monitor | Goal setting, social interaction, badges, progress, rewards | SCT |
| **Non-randomized controlled studies for time spent in MVPA** | | | | |
| Coombes et al.[33] | ** | Activity monitor | Goal setting, feedback, progress, points, rewards, social interaction (social comparison) | Gamification theory |
| **Single group (pre-post) studies for time spent in MVPA** | | | | |
| Fuemmeler et al.[38] | * | Mobile phone: app, activity monitor | Points, badges, levels, story, goal setting, feedback, progress,rewards, challeges, social interaction | SCT, SDT |
| Kouwenhoven-Pasmooij et al.[46] | * | Website, activity monitor | Goal setting, points, rewards, social interaction (competition) | NS |
| Wilson et al.[71] | ^ | Activity monitor (Fuelband) | Goal setting, progress, points, rewards, social interaction | NS |

**Sedentary behavior**

| **Randomized controlled trialss for daily sitting time** | | | | |
| --- | --- | --- | --- | --- |
| Study | Effect | Study modality | Game element | Theory  used |
| Direito et al.[36] | ** | Mobile phone: app (Android and iOS) | Progress, feedback, theme/story (immersive app-Zombies, Run);Progress, feedback(nonimmersive app-Get Running) | Self-regulatory behavior change techniques |
| Pyky et al.[56] | ^ | Activity monitor (wrist-worn) | Feedback, progress, rewards, social interaction(competition) | TTM |
| **Non-randomized controlled studies for daily sitting time** | | | | |
| Yacef et al. [74] | ^ | Mobile phone: app; activity monitor (wrist-worn) | Goal setting, feedback | NS |
| **Single group (pre-post) studies for daily sitting time** | | | | |
| Fuemmeler et al.[38] | * | Mobile phone: app, activity monitor | Points, badges, levels, story, goal setting, feedback, progress,rewards, challeges, social interaction | SCT, SDT |

Notes.**: difference between intervention and control groups is statistically significant; *: pre and post difference within groups is statistically significant; ↑:have a trend but the improvement is not significant; ^: the between group difference or the pre and post difference is not significant. SDT=Self-Determination Theory; COPD= Chronic Obstructive Pulmonary Disease; BCT=Behavior Change Technologies; TPB= Theory of Planned Behavior; HAPA= the Health Action Process Approach; SCT=Social Cognitive Theory; BE= Behavioral Economics; TTM= the Transtheoretical Model; WPWM= the Whole Person Wellness Model.
